# Ultrasensitive negative capacitance phototransistors

**DOI:** 10.1038/s41467-019-13769-z

**Published:** 2020-01-03

**Authors:** Luqi Tu, Rongrong Cao, Xudong Wang, Yan Chen, Shuaiqin Wu, Fang Wang, Zhen Wang, Hong Shen, Tie Lin, Peng Zhou, Xiangjian Meng, Weida Hu, Qi Liu, Jianlu Wang, Ming Liu, Junhao Chu

**Affiliations:** 1 State Key Laboratory of Infrared Physics, Shanghai Institute of Technical Physics, Chinese Academy of Sciences, 500 Yu Tian Road, Shanghai, 200083 China; 2 University of Chinese Academy of Sciences, 19 Yuquan Road, 100049 Beijing, China; 3 Key Laboratory of Microelectronic Devices and Integrated Technology, Institute of Microelectronics, Chinese Academy of Sciences, 100029 Beijing, China; 4 State Key Laboratory of ASIC and System, School of Microelectronics, Fudan University, Shanghai, 200433 China; 5 Hangzhou Institute for Advanced Study, University of Chinese Academy of Sciences, Hangzhou, 310024 China

**Keywords:** Electronic properties and devices, Optical properties and devices, Nanoscale devices

## Abstract

Sensitive photodetection is crucial for modern optoelectronic technology. Two-dimensional molybdenum disulfide (MoS_2_) with unique crystal structure, and extraordinary electrical and optical properties is a promising candidate for ultrasensitive photodetection. Previously reported methods to improve the performance of MoS_2_ photodetectors have focused on complex hybrid systems in which leakage paths and dark currents inevitably increase, thereby reducing the photodetectivity. Here, we report an ultrasensitive negative capacitance (NC) MoS_2_ phototransistor with a layer of ferroelectric hafnium zirconium oxide film in the gate dielectric stack. The prototype photodetectors demonstrate a hysteresis-free ultra-steep subthreshold slope of 17.64 mV/dec and ultrahigh photodetectivity of 4.75 × 10^14^ cm Hz^1/2^ W^−1^ at room temperature. The enhanced performance benefits from the combined action of the strong photogating effect induced by ferroelectric local electrostatic field and the voltage amplification based on ferroelectric NC effect. These results address the key challenges for MoS_2_ photodetectors and offer inspiration for the development of other optoelectronic devices.

## Abstract (web summary)

Here, the authors report ultrasensitive negative capacitance phototransistors based on MoS_2_ regulated by a layer of ferroelectric hafnium zirconium oxide film to demonstrate a hysteresis-free ultra-steep subthreshold slope of 17.64 mV/dec and specific detectivity of 4.75 × 10^14^ cm Hz^1/2^ W^−1^ at room temperature.

## Introduction

Sensitive, fast, and accurate detection of light is the foundation for the future optical communication, memory, sensing, imaging, and other optoelectronic applications. Photodetectors based on two-dimensional (2D) materials, such as graphene^1,2^ and transition metal dichalcogenides^3–5^, have emerged and drawn tremendous attention owing to their unique crystal structures, extraordinary electrical and optical properties, as well as the potential applications in ultrathin, transparent, and flexible optoelectronic devices^6,7^. The nature of a 2D optoelectronic material has largely determined the type and the working mechanism of its photodetectors^8–10^. Taking graphene as an example, as its gapless Dirac cone band structure results in ultrafast carrier recombination, graphene–metal junction and graphene p–n junction photodiodes are mainly exploited for separating photogenerated electrons and holes by the built-in electric field^1,11–13^. Graphene-based photodiodes with the characteristics of fast photoresponse but low photocurrent gain are hardly suitable for the room-temperature few-photon photodetection. On the contrary, molybdenum disulfide (MoS_2_) phototransistors are promising for visible to near-infrared highly sensitive photodetection, due to its thickness-dependent band gap, high photoconductive gain, and high carrier mobility^14–20^. Back in 2013, the monolayer MoS_2_ phototransistor was reported with responsivity (*R*) as high as 880 A W^−1^ at incident wavelength (*λ*) of 561 nm and power of 150 pW^21^. However, since its high responsivity is mainly attributed to trap states either in MoS_2_ or at the interface between MoS_2_ and SiO_2_ substrate, the uncontrollability of trap states caused by material and interface defects, low detectivity, and slow photoresponse on the timescale of seconds seriously limit its practical applications.

Nowadays, nearly all researches on improving the performance of MoS_2_ phototransistors focus on the hybrid systems combined with other material platforms such as graphene, quantum dots (QDs), perovskites, organic dye molecules, and surface plasmonic nanostructures^22^. For instance, the hybrid MoS_2_-PbS QDs photodetector was reported with an ultrahigh responsivity of 6 × 10^5^ A W^−1^, yet large dark current of 0.26 μA, low detectivity, and high operating voltage^23^. Even though hybrid photodetectors using HgTe QDs demonstrate compelling sensitivity, the quality control of QDs and complicated preparation technology remain as the key challenges^24^. Besides, by incorporating organic dye molecules^25^ or perovskites^26^, MoS_2_ photodetectors demonstrate enhanced optical absorption and responsivity, whereas it is inevitable to change the characteristic absorption spectrum and form leakage paths, resulting in large dark current and low detectivity. Furthermore, using high-mobility graphene as an expressway for carrier transport of phototransistors can only improve the performance of MoS_2_ phototransistors to some extent, yet introducing undesirable complex interface and fabrication issues^27^.

Here, an alternative approach is developing gate dielectric engineering in MoS_2_ phototransistors. Ferroelectrics behave nonlinearly, which implies that phototransistors with a ferroelectric layer in the gate stack may have the ability to amplify the electrical response to optical signals. Ferroelectric hafnium zirconium oxide (HZO, Hf_1−x_Zr_x_O_2_ with *x* = 0.5) thin film, with thickness scalability, complementary metal-oxide-semiconductor (CMOS) technology compatibility, and environmental friendliness, is thought as a kind of superior nonlinear gate dielectric layer to regulate optoelectronic characteristics of MoS_2_ phototransistors. Recent researches have demonstrated the photogating effect in MoS_2_ phototransistors, where trapped holes enable photogenerated electrons to circulate through an external circuit more than once leading to large photocurrent^20,28^. Besides, photogating effect can not only achieve the photoconductive gain but also lead to the threshold shift of phototransistors. Although the uncompensated dangling bonds or water molecules at the MoS_2_/oxide interface contribute to trap states to a certain extent, the ferroelectric HZO gate dielectric layer dominates and enhances the photogating effect by trapping more holes in the ferroelectric local electrostatic field, yielding high photoconductive gain and large threshold shifts. Moreover, ferroelectric negative capacitance (NC) effect, that the capacitance of ferroelectrics is negative during the process of ferroelectric polarization switching, possesses the ability to accomplish voltage amplification and ultra-steep subthreshold slope (SS) in the phototransistor with a ferroelectric layer in its gate stack^29–34^. Therefore, MoS_2_ phototransistors based on ferroelectric HZO films can efficiently amplify the change of the channel energy barrier induced by threshold shifts, resulting in that more electrons can go across the channel energy barrier and form large photocurrent. On the whole, ferroelectric HZO film is supposed to improve the performance of MoS_2_ phototransistors through strengthening the photogating effect by trapping more holes in the ferroelectric local electrostatic field and amplifying the energy barrier drop caused by threshold shifts based on ferroelectric NC effect.

In this paper, we report an ultrasensitive NC MoS_2_ phototransistor based on ferroelectric HZO thin film. In contrast to previously reported optimization strategies of MoS_2_ photodetectors, ferroelectric HZO thin films in the gate dielectric stack can significantly enhance the photogating effect, suppress the dark current and improve the light to dark current ratio by ferroelectric local electrostatic field and ferroelectric NC effect, thereby yielding an ultrahigh photodetectivity while maintaining high responsivity and fast response. Experimentally, by the gate structure engineering and the semiconductor thickness scaling^35^, MoS_2_ phototransistors accomplish a hysteresis-free ultra-steep SS (SS_avg_ = 45 mV/dec, SS_min_ = 17.64 mV/dec). When illuminated, transfer characteristic curves shift to the left because of the strong photogating effect induced by ferroelectric local electrostatic field, yielding a change in phototransistor threshold voltage *V*_T_ (in the dark) to *V*_T_ − Δ*V*_T_ (under illumination). As a result of threshold shift, the drain current (*I*_ds_) changes from *I*_dark_ to *I*_light_ = *I*_dark_ + *I*_ph_ and follows *I*_ph_ = *I*_light_ − *I*_dark_ = ∫(d*I*_ds_/d*V*_g_)d*V*_T_, where *I*_ph_ is photocurrent, *I*_dark_ and *I*_light_ are the drain currents in the dark or under illumination. More specially, as d*I*_ds_/d*V*_g_ is fully dependent on SS in the subthreshold region, ultra-steep SS induced by ferroelectric NC effect can efficiently increase photocurrent and improve detectivity^20^. Therefore, based on the combined action of the threshold shift and the ultra-steep SS, we demonstrate the prototype devices with ultrahigh detectivity of 4.7 × 10^14^ cm Hz^1/2^ W^−1^ and high responsivity of 96.8 A W^−1^ under low operating voltages of *V*_ds_ = 0.5 V and *V*_g_ = −1.6 V at room temperature. These results address the key challenges for ultrasensitive MoS_2_ photodetectors and are promising for the development of other photodetectors based on the photogating effect.

## Results

### Device structure and microscopic representation

**Fig. 1:**
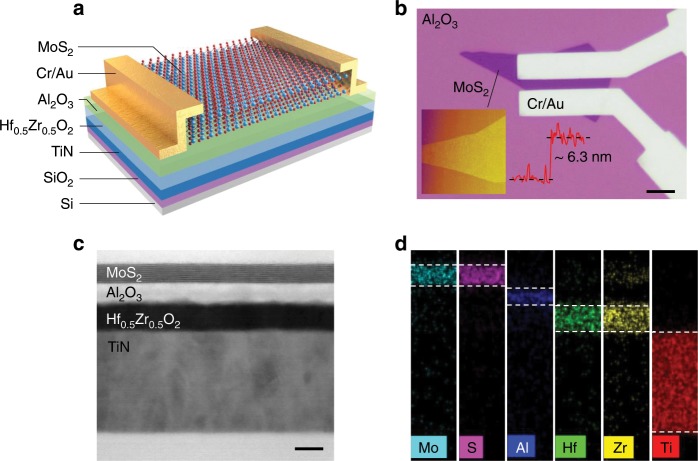
Schematic of device structure and microscopic representation. **a** Structure schematic of MoS_2_ phototransistors with the ferroelectric HZO thin film in the gate dielectric stack. **b** Optical microscope photograph of the device with MoS_2_ channel length of 3 μm, width of 15 μm. Scale bar, 5 μm. The inset is the atomic force microscope (AFM) imaging of the multilayer MoS_2_ flake, and the thickness of MoS_2_ is measured as 6.3 nm (9 layers). **c** Transmission electron microscopy (TEM) imaging of the channel and gate of the device is in order with MoS_2_/Al_2_O_3_/HZO/TiN layers. Interfaces of all layers are clear and flat. Scale bar, 10 nm. **d** Energy dispersive X-ray spectroscopy (EDS) elemental mapping of Mo, S, Al, Hf, Zr and Ti, corresponding to MoS_2_/Al_2_O_3_/HZO/TiN layers in the TEM imaging.

Figure 1a, b is the structure schematic and the optical microscope photograph of the NC MoS_2_ phototransistor, respectively. A metal-ferroelectric-insulator-semiconductor field effect transistor (MFISFET) was fabricated on a p-type silicon substrate, with TiN as the gate metal, HZO/Al_2_O_3_ as ferroelectric/insulator gate layers, multilayer MoS_2_ as the channel semiconductor, and Cr/Au as the source and drain electrodes. The TiN layer is not only used as the back gate electrode but also as the substrate for growing high-quality ferroelectric HZO thin film by atomic layer deposition (ALD) process. The growth process of the ferroelectric HZO thin film is provided in “Methods” section, and the grazing-incident X-ray diffraction (GI-XRD) pattern of the ferroelectric HZO film is shown in Supplementary Fig. 1. Compared with conventional ferroelectrics, ferroelectric HZO thin film with thickness scalability, CMOS compatibility, and environmental friendliness is more applicable in future nanoscale electronic and optoelectronic devices. Besides, the amorphous Al_2_O_3_ layer plays an essential role in improving the quality of HZO/MoS_2_ interface^36^, including suppressing gate leakage current, achieving capacitance matching, and enhancing device stability^31,32^. As shown in Supplementary Fig. 2, metal-ferroelectric-semiconductor field effect transistors (MFSFETs) without the Al_2_O_3_ layer present inferior electrical properties. The inset in Fig. 1b is the atomic force microscope (AFM) imaging of a multilayer MoS_2_ flake, and the thickness of MoS_2_ is measured as 6.3 nm (nine layers) with the Raman spectrum of nine-layer MoS_2_ shown in Supplementary Fig. 3^37^. Figure 1c, d is the cross-sectional transmission electron microscopy (TEM) imaging and the detailed energy dispersive X-ray spectrometry (EDS) elemental mapping of the channel and gate of the device. According to the TEM imaging, the thicknesses of MoS_2_/Al_2_O_3_/HZO/TiN layers are 6.3/6/10/40 nm, and the interfaces of all layers are clear, flat, and free of impurities. Besides, the layered structure of the MoS_2_ layer is discerned in Supplementary Fig. 4. Furthermore, the EDS analysis of Mo, S, Al, Hf, Zr, and Ti elements demonstrates that all elements are uniformly distributed and without inter-diffused.

### Electrical properties of NC MoS_2_ phototransistors

**Fig. 2:**
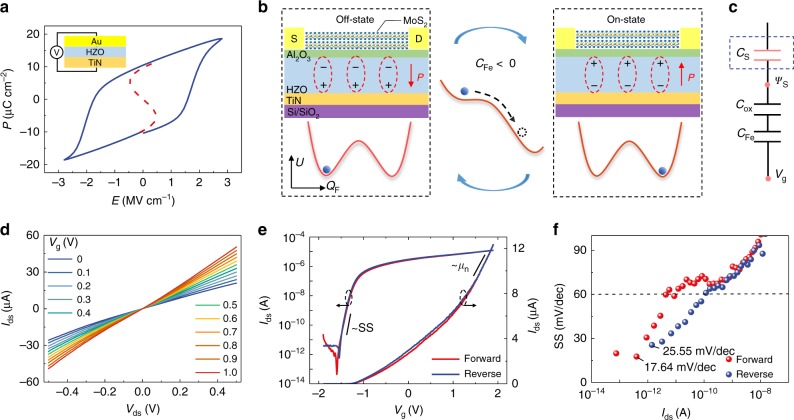
Electrical measurement and results of the NC MoS_2_ phototransistors. **a** Hysteresis loop (solid blue line) of the 10 nm-thick ferroelectric HZO thin film is measured by a capacitor composed of Au/HZO/TiN layers. The dashed red line fitted by the Landau–Khalatnikov (LK) equation *E* = 2*αP* + 4*βP*^3^ + 6*γP*^5^ shows negative d*P*/d*E* region. **b** Device working schematics and ferroelectric double well energy landscapes. During the process of ferroelectric polarization switching, the ferroelectric material is supposed to go across an energy barrier from one energy valley to another. This energy barrier is regarded as the ferroelectric NC region. **c** Equivalent capacitance schematic of the device, where *C*_S_, *C*_ox_ and *C*_Fe_ represent the capacitance of MoS_2_, Al_2_O_3_ and HZO layers, respectively. **d** Output characteristic (*V*_ds_–*I*_ds_) curves exhibit ohmic characteristics for all gate voltages. **e** Forward and reverse transfer characteristic (*V*_g_–*I*_ds_) curves under *V*_ds_ = 0.1 V are plotted in red and blue, respectively, and hysteresis between them is almost negligible. Electron mobility and SS are, respectively, extracted based on a linear scale (right axis) and a logarithmic scale (left axis). **f** SS is calculated according to the transfer characteristic curves, where the average value, the minimum values in forward and reverse (SS_avg_, SS_min-for_, and SS_min-rev_) are 45, 17.64, and 25.55 mV/dec, respectively.

The ferroelectric properties of the HZO thin film, such as the hysteresis loop and the NC effect, play decisive roles in electrical and optoelectronic characteristics of NC phototransistors. Firstly, the solid blue line in Fig. 2a presents the hysteresis loop of the 10 nm-thick ferroelectric HZO thin film tested by a capacitor composed of Au/HZO/TiN layers (the maximum swept voltage of the *P*-*E* measurement is ±2.8 V, the frequency used is 1000 Hz, and the preset delay is 1000 ms). The hysteresis loop confirms the basic ferroelectric characteristics of HZO films with coercive field (*E*_c_) of 1.5 MV cm^−1^ and remnant polarization (*P*_r_) of 10 μC cm^−2^, and the dashed red line fitted by the Landau–Khalatnikov (LK) equation *E* = 2*αP* + 4*βP*^3^ + 6*γP*^5^ shows the negative d*P*/d*E*^29,32^. Furthermore, the ferroelectric capacitance (*C*_Fe_) is defined as the reciprocal of the second derivation of the potential energy (*U)* with respect to the charge (*Q)*, written as *C*_Fe_ = (d^2^*U*/d*Q*^2^)^−1^. The principle of the ferroelectric NC effect can be understood through device working mechanism schematics and ferroelectric double well energy landscapes^34^ shown in Fig. 2b. At the beginning, the gate voltage of the device is negative and less than the threshold voltage, yielding the ferroelectric layer polarized downwards, the channel electrons depleted, and the device turned off. Then changing the gate voltage to the opposite, the ferroelectric layer is polarized upwards, the channel electrons are accumulated, and the device is turned on. In the process of mutual conversion of the above two ferroelectric polarization states, the ferroelectric is supposed to go across an energy barrier from one energy valley to another. According to the definition of the ferroelectric capacitance, this energy barrier is the ferroelectric NC region.

To get insight into the fundamental electrical characteristics of the NC MoS_2_ phototransistors, Fig. 2c exhibits its equivalent capacitance schematic, where *C*_S_, *C*_ox_, and *C*_Fe_ represent the capacitance of MoS_2_, Al_2_O_3_, and HZO layers, respectively. Notably, *C*_S_ also includes the stray capacitances of the source and drain shown in Supplementary Fig. 5. *C*_ox_^38^ and *C*_Fe_ can be calculated by the expression *C* = *εSd*^−1^, where *ε*, *S*, and *d* stand for the permittivity, area and thickness of dielectric layers, and the permittivity of the ferroelectric HZO thin film (*ε*_Fe_) presented in Supplementary Fig. 6 is measured by the capacitor shown in Fig. 2a. Through a simple calculation, the relationship between SS and capacitances is obtained as SS = [1 + *C*_S_(*C*_Fe_^−1^ + *C*_ox_^−1^)] × 60 mV/dec = [1 − *C*_S_(|*C*_Fe_|^−1^ − *C*_ox_^−1^)] × 60 mV/dec. According to this expression, it is beyond doubt that capacitance matching is crucial to achieve sub-60 mV/dec ultra-steep SS. Based on an optimized 10 nm-thick HZO ferroelectric layer and an appropriate 6 nm-thick Al_2_O_3_ layer (according to the expression of SS, |*C*_Fe_|^−1^ − *C*_ox_^−1^ > 0 is a necessary condition for the sub-60 mV/dec ultra-steep SS), a series of thicknesses of MoS_2_ are measured experimentally and the measurement results are shown in Supplementary Fig. 7. As the thickness of MoS_2_ increases, that is the permittivity and capacitance of MoS_2_ increase^35^, the SS becomes steeper in accordance with the expression SS = [1 − *C*_S_(|*C*_Fe_|^−1^ − *C*_ox_^−1^)] × 60 mV/dec. Meanwhile, when the thickness of the MoS_2_ is increased to 9.8 nm, a hysteresis about 0.16 V between forward and reverse *V*_g_–*I*_ds_ curves is observed, which can be explained by the structural defects of MoS_2_ or the stability condition of NCFETs. For one thing, the total number of structural defects increases with the thickness of MoS_2_^39,40^. When *V*_g_ sweeps forward, charge traps in the MoS_2_ layer inhibit the ferroelectric polarization switching by screening the electric field. After the traps have been neutralized, the ferroelectric polarization switches, leading to a large hysteresis and an abrupt jump in the reverse. For another, the stability condition of MFISFETs requires that the total gate-channel capacitance *C*_gc_ = (*C*_S_^−1^ + *C*_ox_^−1^ + *C*_Fe_^−1^)^−1^ = (*C*_S_^−1^ + *C*_ox_^−1^ − |*C*_Fe_|^−1^)^−1^ has to be positive^29,31^. Increasing the thickness (capacitance) of MoS_2_ may break the stability condition of the device, resulting in a large hysteresis in *I*_ds_–*V*_g_ curves.

Through optimizing the thickness of MoS_2_ in MFISFETs to 6.3 nm (nine layers), the MoS_2_ phototransistor accomplishes hysteresis-free ultra-steep SS. The output characteristic (*V*_ds_–*I*_ds_) curves shown in Fig. 2d exhibit ohmic characteristics for all gate voltages. The output characteristic curves under larger drain voltages are presented in Supplementary Fig. 8. In Fig. 2e, the forward and reverse transfer characteristic (*V*_g_–*I*_ds_) curves (the swept voltage of *V*_g_–*I*_ds_ measurements is ramped and the swept rate is 14.14 Hz corresponding to the number of data points per second) are plotted in red and blue, respectively, and the hysteresis between them is almost negligible (the gate leakage measurement is shown in Supplementary Fig. 9). For a linear scale (right axis), electron mobility (*μ*_n_) is calculated as 54.2 cm^2^ V^−1^ s^−1^ using the expression *μ*_n_ = (*L*/*W*)(*S*/*C*_ins_)*V*_ds_^−1^(d*I*_ds_/d*V*_g_), where *C*_ins_ stands for the total capacitance of gate insulators. For a logarithmic scale (left axis), SS calculated by the definition SS = d*V*_g_/d(lg*I*_ds_) are presented in Fig. 2f, where the average value, the minimum value in forward and reverse (SS_avg_, SS_min-for_, and SS_min-rev_) are 45, 17.64, and 25.55 mV/dec, respectively. The optimization of electrical characteristics paves the way for ultrasensitive photodetection of MoS_2_ phototransistors working in the subthreshold region.

### Mechanism of optical detection in NC MoS_2_ phototransistors

**Fig. 3:**
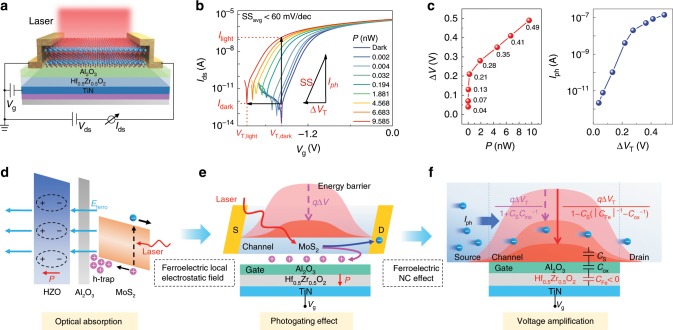
Mechanism of ultrasensitive optical detection in the NC MoS_2_ phototransistors. **a** Laser is incident perpendicularly to the device surface, and the spot covers the entire channel uniformly. **b** *V*_g_–*I*_ds_ curves at *V*_ds_ = 0.5 V in the dark and under various incident light powers (*λ* = 520 nm). **c** Dependence of threshold shifts on the incident optical powers and dependence of photocurrent on threshold shifts are extracted from *V*_g_–*I*_ds_ curves. **d** Under light illumination, enhanced light absorption can be achieved in multilayer MoS_2_, generating electron-hole pairs efficiently by a strong light-matter interaction. **e** Photoexcited electrons are pulled toward the electrodes, while photoexcited holes are trapped in the gate stack due to the ferroelectric electrostatic local field, leading to strong photogating effect on the phototransistors. Photogating effect gives rise to a threshold shift as well as a reduction of the energy barrier in the channel (*q*Δ*V*), so that more electrons can get over the energy barrier and forms photocurrent. **f** In generally, *q*Δ*V* *=* *q*Δ*V*_T_(1 + *C*_S_*C*_ins_^−1^)^−1^ < *q*Δ*V*_T_ since the total capacitance of gate insulators (*C*_ins_) is usually positive. However, MoS_2_ NC phototransistors can break through this limitation and achieve a larger *q*Δ*V* *=* *q*Δ*V*_T_[1 − *C*_S_(|*C*_Fe_|^−1^ − *C*_ox_^−1^)]^−1^ > *q*Δ*V*_T_ based on the ferroelectric NC effect and capacitance matching conditions.

For typical photodetection operation, the laser is incident perpendicularly to the device surface, and the spot covers the entire channel uniformly shown in Fig. 3a. The transfer characteristics of the phototransistors in the dark and under the illumination of various optical powers are presented in Fig. 3b. The changes in threshold voltage *V*_T,dark_ (in the dark) to *V*_T,light_ = *V*_T_ − Δ*V*_T_ (under illumination) are observed in *V*_g_–*I*_ds_ curves, and the threshold shift (Δ*V*_T_) increases with the incident optical powers. As a result of the threshold shift, the channel current increases from *I*_dark_ to *I*_light_ = *I*_dark_ + *I*_ph_, that is the photocurrent *I*_ph_ = *I*_light_ − *I*_dark_ = ∫(d*I*_ds_/d*V*_g_)d*V*_T_, where d*I*_ds_/d*V*_g_ is fully dependent on SS in the subthreshold region. According to the definition of SS, with simple algebra the quantitative relationship among Δ*V*_T_, *I*_ph_ and SS is obtained as lg(*I*_light_/*I*_dark_) = lg*I*_light_ − lg*I*_dark_ = ∫SS^−1^d*V*_T_, indicating that the large threshold shift and the ultra-steep SS work together to contribute to the ultrahigh light to dark current ratio and achieve ultrasensitive photodetection. Dependence of threshold shifts on incident optical powers and dependence of photocurrent on threshold shifts are extracted from *V*_g_–*I*_ds_ curves and presented in Fig. 3c. Since the ability of the ferroelectric local electrostatic field to trap holes is gradually weakened as holes accumulated more and more in the gate stack, *P*–Δ*V*_T_ and Δ*V*_T_–*I*_ph_ curves rise steeply for the incident optical power up to 0.2 nW and the threshold shift up to 0.21 V, respectively, and then gradually saturates.

To explore the mechanism of ultrasensitive photodetection, we focus on the working principles of optical absorption, photogating effect and voltage amplification in the device. Throughout the entire photodetection process, the operating voltages *V*_ds_ and *V*_g_ are set as constant, and the gate voltage equals to the threshold voltage of the device in the dark. For the dark state, the ferroelectric layer is polarized by the gate voltage, and the channel electrons are depleted by the ferroelectric electrostatic local field, therefore the phototransistor is turned off, and the dark current is suppressed to an extremely low level. Under light illumination, the enhanced light absorption can be achieved in multilayer MoS_2_, generating electron-hole pairs efficiently by a strong light-matter interaction shown in Fig. 3d. Crucially, photoexcited electrons are pulled toward the external circuits, while photoexcited holes are trapped in the gate stack due to the ferroelectric electrostatic local field (*E*_ferro_), leading to a strong photogating effect on the MoS_2_ phototransistors. Furthermore, as presented in Fig. 3e, the photogating effect gives rise to a change of threshold voltage (Δ*V*_T_) as well as a reduction of the energy barrier (*q*Δ*V*) in the channel, so that more electrons can get over the energy barrier and form photocurrent. As shown in Fig. 3f, the channel energy barrier drop *q*Δ*V* *=* *q*Δ*V*_T_(1 + *C*_S_*C*_ins_^−1^)^−1^ is generally less than *q*Δ*V*_T_, since the total capacitance of gate insulators is usually positive. However, MoS_2_ NC phototransistors can break through this limitation and achieve a larger *q*Δ*V* *=* *q*Δ*V*_T_[1 − *C*_S_(|*C*_Fe_|^−1^ − *C*_ox_^−1^)]^−1^ > *q*Δ*V*_T_ based on the ferroelectric NC effect and capacitance matching conditions. It means that ultrasensitive photodetection of NC MoS_2_ phototransistors is fundamentally based on the combined action of the ferroelectric electrostatic local field and the ferroelectric NC effect.

### Optoelectronic properties of NC MoS_2_ phototransistors

**Fig. 4:**
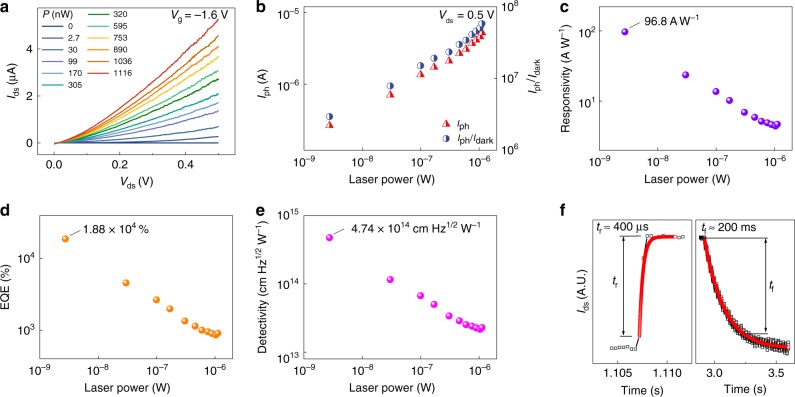
Optoelectronic measurement and results of the NC MoS_2_ phototransistors. **a** *V*_ds_–*I*_ds_ curves in the dark and under various incident optical powers (*λ*  = 637 nm). **b** With the logarithmic scale, the photocurrent (in red) and the light to dark current ratio (in blue) increase with the incident powers and achieve maximum values as high as *I*_ph_ = 5.22 μA and *I*_light_/*I*_dark_ = 5.8 × 10^7^ at *P*_eff_ = 1116 nW. **c**, **d**, **e** Responsivity *R* = *I*_ph_*P*_eff_^−1^, external quantum efficiency EQE = *hcRλ*^−1^*e*^−1^ and detectivity D* = *RA*^1/2^(2*eI*_dark_)^−1/2^ show the same changing tendency with the incident powers, reaching the largest values of *R* **=** 96.8 A W^−1^, EQE = 1.88 × 10^4^ %, D* = 4.75 × 10^14^ cm Hz^1/2^ W^−1^ at the lowest incident power of *P*_eff_ = 2.7 nW. **f** Rise (*τ*_r_) and decay (*τ*_f_) times of photocurrent are measured as 400 μs and 200 ms, respectively, and both the rise and decay curves (in black) are fitted well using exponential functions (in red).

To confirm the above mechanism, we next turn our attention to the photoresponse of the NC MoS_2_ phototransistors. The optoelectronic measurements are performed under the illumination of a monochromatic light source with wavelength of 637 nm in a dark room at room temperature. Figure 4a presents the *V*_ds_–*I*_ds_ curves in the dark and under various incident optical powers. To further evaluate the performance of the phototransistors, we extract the power dependence of photocurrent, light to dark current ratio, responsivity, external quantum efficiency (EQE) and detectivity from the *V*_ds_–*I*_ds_ curves at *V*_ds_ = 0.5 V and *V*_g_ = −1.6 V. With the logarithmic scale, the photocurrent dotted in red and the light to dark current ratio dotted in blue increase with the incident optical powers and achieve maximum values as high as *I*_ph_ = 5.22 μA and *I*_light_/*I*_dark_ = 5.8 × 10^7^ at *P*_eff_ = 1116 nW as shown in Fig. 4b. It is worth mentioning that *P*_eff_ is calculated by *P*_eff_ = *AP*π^−1^*r*^−2^, where *A* is the area of the sample, *P* is the actual laser output and *r* is the radius of the laser spot size. The responsivity *R* = *I*_ph_*P*_eff_^−1^ indicates how much photocurrent can be produced by unit incident power on the photodetector, while the EQE defined as EQE = *hcRλ*^−1^*e*^−1^, is the ratio of the number of photoexcited carriers to the number of incident photons, where *h* is Planck constant, *c* is the speed of light and *e* is the electron charge. The responsivity and the EQE respectively plotted in Fig. 4c, d show the same changing tendency with the incident powers, reaching the largest values of *R* **=** 96.8 A W^−1^ and EQE = 1.88 × 10^4^ % at the lowest incident power of *P*_eff_ = 2.7 nW. The detectivity given by D* = *RA*^1/2^(2*eI*_dark_)^−1/2^ is one of the most important parameters for photodetectors. The ultrahigh detectivity of D* = 4.75 × 10^14^ cm Hz^1/2^ W^−1^ is achieved at *P*_eff_ = 2.7 nW in Fig. 4e owing to the ultrahigh light to dark current ratio. Moreover, rise (*τ*_r_) and decay (*τ*_f_) times of photocurrent are measured as 400 μs and 200 ms, respectively, and both the rise and decay curves (in black) are fitted well using exponential functions (in red) shown in Fig. 4f.

**Fig. 5:**
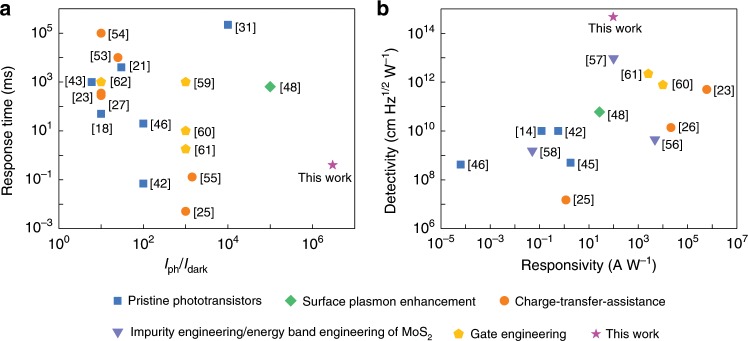
Optimization strategy and performance summary of reported MoS_2_ phototransistors. Optoelectronic properties (**a** response time and the ratio of photocurrent to dark current. **b** responsivity and detectivity) of NC MoS_2_ phototransistors based on ferroelectric HZO gate dielectric engineering in this work outperform most of the previously reported MoS_2_ phototransistors based on other strategies regarding methods, including surface plasmon enhancement, charge-transfer assistance and impurity/energy band engineering of MoS_2_.

We have also reviewed methods to improve the performance of MoS_2_ phototransistors based on previously reported researches and this work. As one kind of the most investigated 2D material-based photodetectors, optimization strategies and detailed performance parameters of MoS_2_ phototransistors are listed in Supplementary Table 1 to provide ideas for further exploration of other 2D optoelectronic semiconductors and their photodetectors^41–62^. There are four kinds of strategies regarding methods, including surface plasmon enhancement, charge-transfer assistance, impurity/energy band engineering of MoS_2_, and gate engineering. Among these methods, charge-transfer-assisted hybrid systems combined with other material platforms such as graphene, QDs, perovskites and organic molecules are the most extensively studied strategy recently. The hybrid phototransistors present enhanced optical absorption and responsivity, whereas it is usually inevitable to introduce leakage paths, increase dark current, and slower response speed. As presented in Fig. 5, NC MoS_2_ phototransistors based on gate dielectric engineering can circumvent these issues and demonstrate compelling performance with ultrahigh light to dark current ratio and detectivity, high responsivity and fast response, are promising for low-power and high-performance optoelectronic applications.

## Discussion

In summary, based on the strategy of gate dielectric engineering, NC MoS_2_ phototransistors with HZO ferroelectric thin films in the gate dielectric stack are proposed and fabricated. The prototype photodetectors demonstrate ultra-steep subthreshold slope of 17.64 mV/dec and ultrahigh detectivity of 4.75 × 10^14^ cm Hz^1/2^ W^−1^ under *λ* = 637 nm and *P*_eff_ = 2.7 nW at room temperature, owing to the strong photogating effect of ferroelectric local electrostatic field and the ultra-steep SS induced by ferroelectric NC effect during the process of ferroelectric polarization switching. This high-performance MoS_2_ photodetector should be a promising candidate for future optoelectronic applications and offer inspiration for the development of other optoelectronic devices. Furthermore, the gain mechanism relying on the combined action of the photogating and NC effects can be further exploited for the ultrasensitive few-photon detection technique and generally applicable to a large amount of low-dimensional semiconductors.

## Methods

### Fabrication of the multilayer MoS_2_ phototransistor

A 40 nm-thick TiN layer was deposited by ion beam sputtering on 300 nm-thick SiO_2_ grown on p-doped silicon substrates. The 10 nm-thick HZO thin film was grown on the TiN gate electrode by ALD process at 280 °C substrate temperature, and crystalized with a 60 s anneal at 500 °C in a nitrogen atmosphere. Hf[N(C_2_H_5_)CH_3_]_4_, Zr[N(C_2_H_5_)CH_3_]_4_ and H_2_O were used as Hf precursor, Zr precursor and oxygen source, respectively. The Hf/Zr ratio was controlled by alternate deposition of one cycle HfO_2_ and one cycle ZrO_2_, which was confirmed by X-ray photoelectron spectroscopy (XPS). Then a 6 nm-thick Al_2_O_3_ layer was deposited on the HZO film by electron beam evaporation. Multilayer MoS_2_ nano-sheets were mechanically exfoliated from bulk MoS_2_ crystals and mounted on the Al_2_O_3_ layer. Finally, the source (S) and drain (D) metal electrodes were fabricated using electron-beam lithography, thermal evaporation of Cr/Au films (15 nm / 45 nm) and lift-off.

### Polarization of the ferroelectric HZO Film

In MoS_2_ NC phototransistors, the source electrode is connected to ground, and the polarization state of the ferroelectric HZO film in the gate stake is controlled by the gate voltage *V*_g_, specific as follows. For the electrical measurement, taking Fig. 2e as an example, during the *I*_ds_–*V*_g_ measurement (*I*_g_–*V*_g_ is measured simultaneously and the results are shown in Supplementary Fig. 9), the source electrode is connected to ground, the drain voltage is constant (*V*_ds_ = 0.1 V), and the gate voltage sweeps forward (*V*_g_ is from –1.9 to 1.9 V) and then reverse (from 1.9 to −1.9 V). As *V*_g_ sweeps forward, the ferroelectric polarization switches from downward to upward, the channel electrons are gradually accumulated and the device is turned on. As *V*_g_ sweeps reverse, the ferroelectric polarization switches from upward to downward, the channel electrons are gradually depleted and the device is turned off. For the optoelectronic measurement, taking Fig. 4a as an example, the source electrode is connected to ground, the operating voltages *V*_ds_ and *V*_g_ are set as constant, and specially the gate voltage equals to the threshold voltage of the dark state (*V*_g_ = *V*_T,dark_ = −1.6 V), yielding that the ferroelectric layer is polarized downwards and the dark current is suppressed to an extremely low level.

### Measurement of electrical and optoelectronic properties

The electrical and optoelectronic measurement were carried out with a Lake Shore probe station and a Keysight B1500A semiconductor device analyzer under ambient conditions at room temperature. The photoresponse was tested under illumination of 520 nm/637 nm monochromatic light sources in a dark room. The light source was generated, switched, and transmitted by a Thorlabs ITC4001 laser diode/temperature controller and a LM9LP fiber-pigtailed laser diode. The spot diameter of the light source on the device surface was 1 mm, and the multilayer MoS_2_ nano-sheet of the device was positioned at the center of the spot. A high-speed Tektronix MDO3014 mixed domain oscilloscope was used to measure the time-resolved photoresponse.

## Supplementary information

Supplementary Information

## Data Availability

The data that support the plots within this paper and other findings of this study are available from the corresponding author upon reasonable request. The source data underlying Figs. 2a, d–f, 3b, c, 4a–f, 5a, b and Supplementary Figs. 1, 2b–d, 3, 6, 7b, c, e, f, 8 and 9 are provided as a Source Data file.

## Source data

Source Data
