# Supplementary material for: Ultrasensitive negative capacitance phototransistors
Source: Nat Commun. 2020 Jan 3;11:101. doi: 10.1038/s41467-019-13769-z (PMC6941953; doi:10.1038/s41467-019-13769-z)
Supplement: Supplementary file 1 — Supplementary Information [file 41467_2019_13769_MOESM1_ESM.pdf]

Supplementary Information for
Ultrasensitive Negative Capacitance Phototransistors

Tu et al.

Supplementary Figure 1

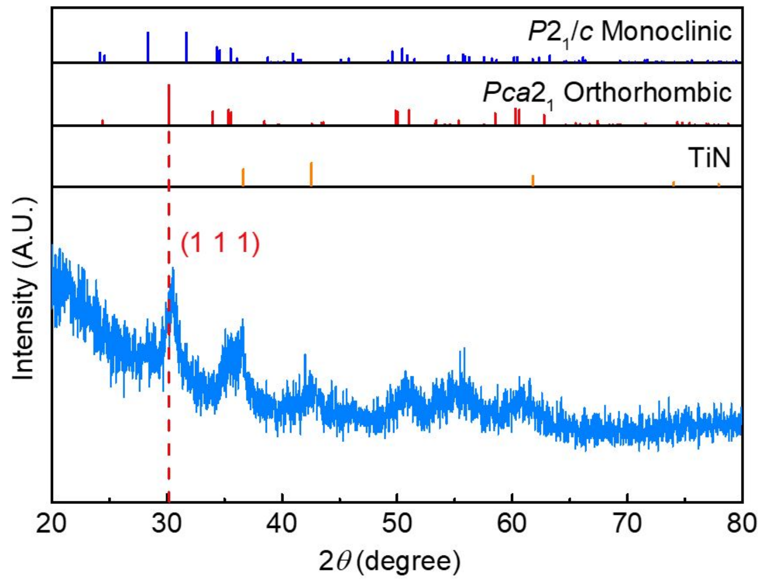

Supplementary Fig. 1 | GI-XRD pattern of the ferroelectric HZO film. The grazing-incident X-ray diffraction (GI-XRD) pattern of the 10 nm-thick HZO thin film at an incident angle of 1° is well matched with the ferroelectric orthorhombic phase *Pca*2_1 (with the highest peak at 30.2° corresponding to the 111_o reflection)[1-3].

Supplementary Figure 2

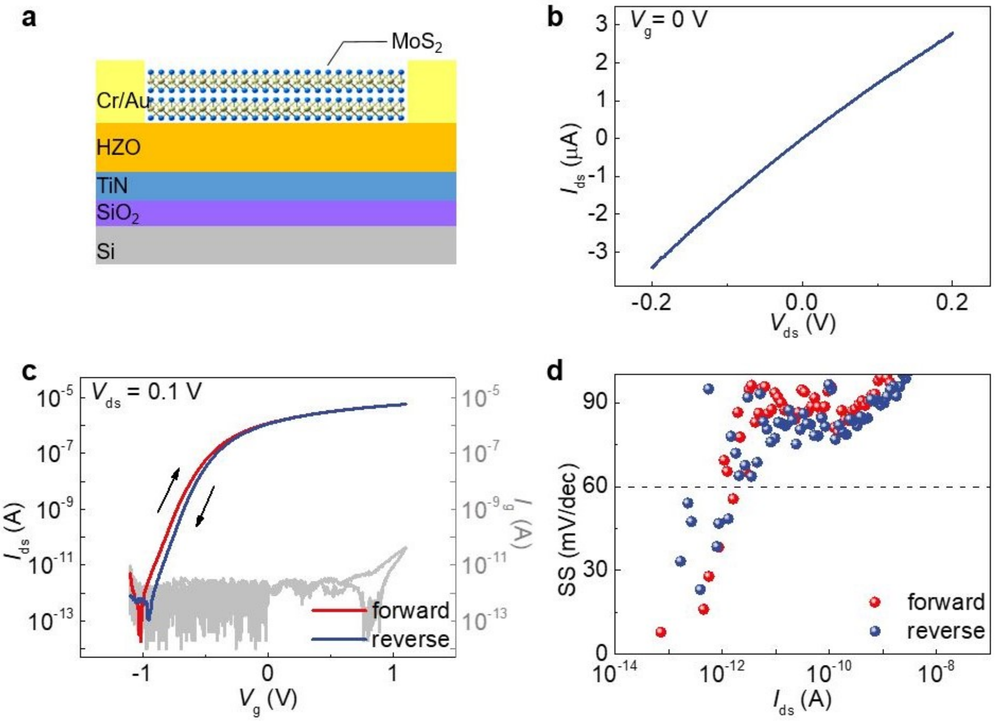

Supplementary Fig. 2 | Device schematic and electrical properties of MFSFETs. a, Device schematic of a metal-ferroelectric-semiconductor field effect transistor (MFSFET). **b**, Output characteristic (V_{ds}-I_{ds}) curve exhibits ohmic characteristics. **c**, Forward and reverse transfer characteristic (V_g-I_{ds}) curves are plotted in red and blue respectively. **d**, SS is calculated according to the transfer characteristic curves.

The comparison results of the electronic properties between the MFSFET and the MFISFET are listed as follows: 1) As the gate voltage sweeps from negative to positive and then to negative, the V_g-I_{ds} curve of the MFSFET exhibits a clockwise hysteresis, while the MFISFET is almost non-hysteresis. This phenomenon is due to the higher interface defect density in the HZO/MoS_2 interface of the MFSFET without an Al_2O_3 buffer layer. 2) The MFSFET without the Al_2O_3 layer presents the higher SS than the MFISFET (the MFISFET presents the sub-60 mV/dec SS over three orders of magnitude in drain current, while that is over just one order of magnitude in the MFSFET). In addition, when the gate voltage V_g is more than 1V, the gate of the MFSFET has a more obvious leakage trend than the MFISFET.

Supplementary Figure 3

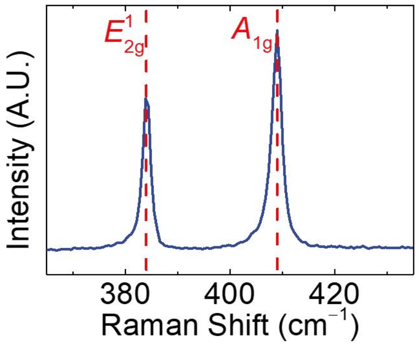

Supplementary Fig. 3 | Raman spectrum of the 9-layer MoS_2 flake. Raman spectrum of the 9-layer MoS_2 flake with peaks at Raman frequencies of 383.87 cm^{-1} (E^1_{2g}) and 408.97 cm^{-1} (A_{1g}).

Supplementary Figure 4

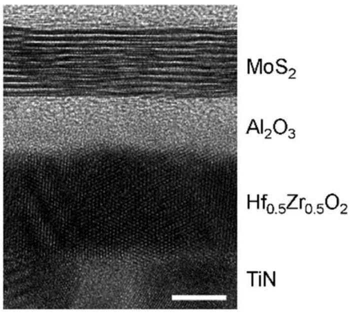

Supplementary Fig. 4 | High-resolution TEM imaging of the device. High-resolution cross-sectional transmission electron microscopy (TEM) imaging of the device, where the layered structure of the MoS_2 flake is discerned. Scale bar, 5 nm.

Supplementary Figure 5

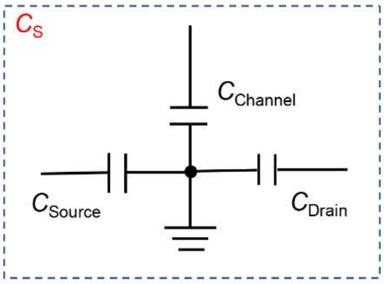

Supplementary Fig. 5 | Equivalent capacitance schematic. Semiconductor capacitance (C_S) includes capacitance of MoS_2 as well as stray capacitances of the source and drain (C_{Source} and C_{Drain}).

Supplementary Figure 6

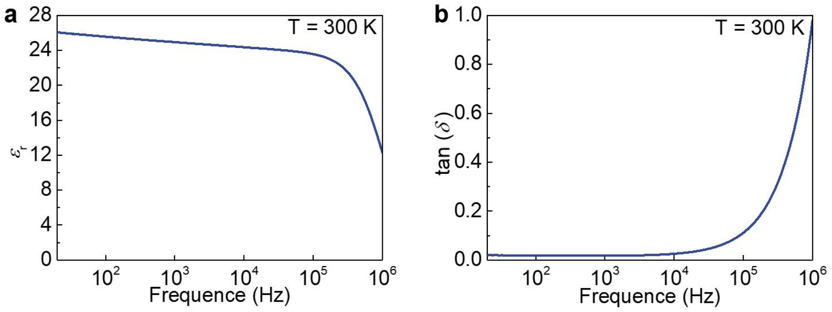

Supplementary Fig. 6 | Relative permittivity and dielectric loss of the ferroelectric HZO film. Capacitance of the ferroelectric HZO film (C_{Fe}) is calculated by $C_{Fe} = \varepsilon_{Fe} S d_{Fe}^{-1}$, where ε_{Fe}, S and d_{Fe} stand for the permittivity, area and thickness of the ferroelectric HZO film. **a**, Relative permittivity (ε_r) and **b**, dielectric loss of the ferroelectric HZO film are measured by a capacitor composed of Au/HZO/TiN layers.

Supplementary Figure 7

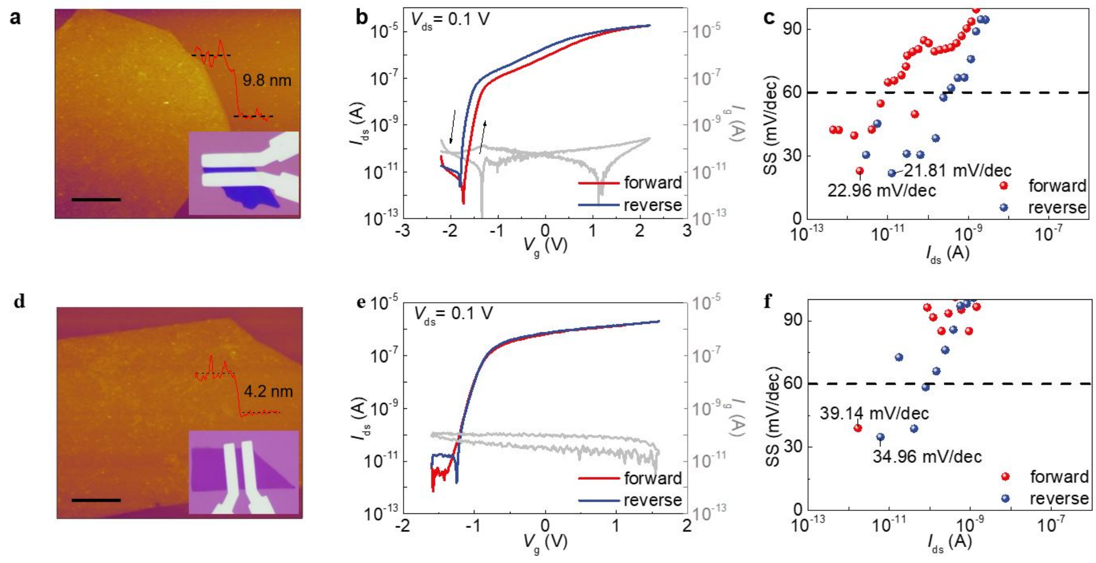

Supplementary Fig. 7 | Electrical properties of MFISFETs with various MoS_2 thicknesses. For a 9.8 nm-thick MoS_2 flake, **a**, AFM imaging of the MoS_2 flake, and the inset is the optical microscope photograph of the device. **b**, a hysteresis about 0.16 V between forward and reverse transfer characteristic (V_g-I_{ds}) curves is observed. **c**, SS is calculated according to V_g-I_{ds} curves, where minimum values in forward and reverse are 22.96 and 21.81 mV/dec. For a 4.2 nm-thick MoS_2 flake, **d**, AFM imaging of the MoS_2 flake and optical microscope photograph of the device. **e**, Hysteresis between forward and reverse transfer characteristic curves is nearly disappeared. **f**, Minimum SS in forward and reverse are 39.14 and 34.96 mV/dec.

Supplementary Figure 8

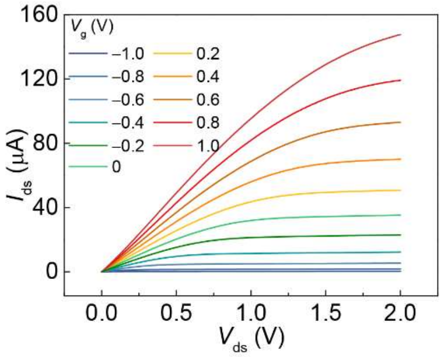

Supplementay Fig. 8 | Output characteristics of the MFISFET. Output characteristic (V_{ds}-I_{ds}) curves under the maximum drain voltage to 2.0 V for V_g from −1.0 to 1.0 V.

Supplementary Figure 9

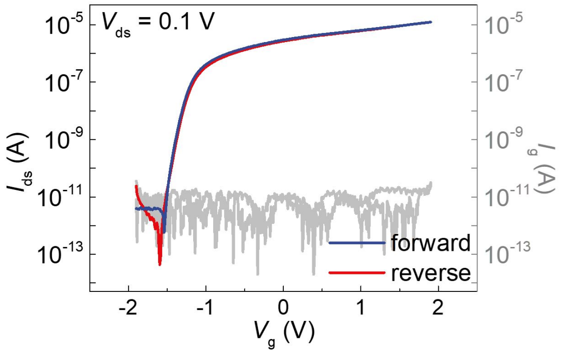

Supplementary Fig. 9 | Gate leakage measurement of the MFISFET. The gate leakage current I_g is well suppressed, which demonstrates that the device is not driven by a leakage behavior.

Supplementary Table 1

Strategy	Device type	Active layer	I_{ph} / I_{dark}	R (AW^{-1})	D^* (cmHz$^{1/2}$W^{-1})	Response time	Operating voltage	Year	Ref.
Pristine phototransistors	Pristine phototransistors based on mechanically exfoliated MoS_2	Mechanically exfoliated 1L	10	7.5×10^{-3}	-	τ_r = 50 ms<br>τ_f = 50 ms	V_{ds} = 1.0 V<br>V_g = 50 V	2011	[4]
		Mechanically exfoliated ML (UV-visible-NIR)	< 10^3	0.12	10^{10}-10^{11}	-	V_{ds} = 1.0 V<br>V_g = −3.0 V	2012	[5]
		Mechanically exfoliated 1L	30	880	-	τ_r = 4 s<br>τ_f = 9 s	V_{ds} = 8.0 V<br>V_g = −70 V	2013	[6]
	Pristine phototransistors based on CVD-grown MoS_2	CVD-grown 1L (in vacuum)	10^4	2.2×10^3	-	220 s	V_{ds} = 1.0 V<br>$V_g - V_{th}$ = 100 V	2013	[7]
		CVD-grown FL	10^2	0.57	10^{10}	τ_r = 70 μs<br>τ_f = 110 μs	V_{ds} = 10 V	2013	[8]
		CVD-grown 1L	10^4	1.1×10^{-3}	-	> 1 s	V_{ds} = 1.5 V<br>V_g = −3.0 V	2014	[9]
	Pristine phototransistors of MoS_2 based on other preparation methods	Self-limiting grown FL (after laser micromachining)	-	0.55	-	τ_r = 200 μs<br>τ_f = 1.7 ms	V_{ds} = 3.0 V<br>V_g = 0 V	2014	[10]
		Magnetron sputtered 5L	-	1.8	5×10^8	τ_r = 0.3 s<br>τ_f = 0.36 s	V_{ds} = 5.0 V	2015	[11]
		Solution-synthesized 2-4L	10^2	6.3×10^{-5}	4.2×10^8	20 ms	V_{ds} = 10 V<br>V_g = 0 V	2016	[12]
Surface plasmon enhancement	Phototransistors with Au nanostructure arrays	Mechanically exfoliated FL	< 10	-	-	-	V_{ds} = 1.0 V	2015	[13]
	Phototransistors with Au Schottky junction	Mechanically exfoliated ML	10^3-10^5	26.9	6×10^{10}	τ_r = 645 ms<br>τ_f = 584 ms	V_{ds} = 1.0 V<br>V_g = −20 V	2019	[14]
Charge-transfer assistance	MoS_2 - QDs phototransistors	FL MoS_2 - PbS QDs (Visible-NIR)	< 10	6×10^5	5×10^{11}	τ_f = 350 ms	V_{ds} = 1.0 V<br>V_g = −60 V	2014	[15]
		FL MoS_2 - PbSe QDs (NIR)	-	1.9×10^{-6}	-	τ_r = 250 ms<br>τ_f = 430 ms	V_{ds} = 14 V	2014	[16]
		ML MoS_2 - Gr QDs	-	1.6×10^4	-	τ_r = 1.23 s<br>τ_f = 10.97 s	V_{ds} = 1.0 V<br>V_g = 80 V	2015	[17]
		MoS_2 - HgTe QDs	-	10^6	-	-	V_{ds} = 1.0 V<br>V_g = 0 V	2017	[18]
	MoS_2 - organic molecules phototransistors	1L MoS_2 - rhodamine 6G (Visible-NIR)	< 10	1.17	1.5×10^7	τ_r = 5.1 μs<br>τ_f = 2.3 s	V_{ds} = 5.0 V<br>V_g = 0 V	2014	[19]
		1L MoS_2 - ZnPc molecules	-	430	-	< 8 ms	V_{ds} = 1.6 V<br>V_g = 40 V	2018	[20]
	MoS_2 - perovskite phototransistors	Solution-processed MoS_2 - $CH_3NH_3PbI_3$ perovskite	-	10^3	2.6×10^{11}	τ_r = 25 ms<br>τ_f = 50 ms	V_{ds} = 2.0 V	2016	[21]
		APTES doped FL MoS_2 - $CH_3NH_3PbI_3$ perovskite	10	2.11×10^4	1.38×10^{10}	τ_r = 10.7 s<br>τ_f = 6.0 s	V_{ds} = 5.0 V<br>V_g = 20 V	2016	[22]
	MoS_2 - Gr phototransistors	ML MoS_2 - 1L Gr	25	5×10^8	-	Tens of seconds	V_{ds} = 0.1 V<br>V_g = −50 V	2013	[23]
		1L MoS_2 - 1L Gr	< 10	1.2×10^7	-	Hundreds of seconds	V_{ds} = 1.0 V<br>V_g = −10 V	2014	[24]
		FL MoS_2 - 1L Gr	< 10	10	-	τ_r = 0.28 s<br>τ_f = 1.5 s	V_{ds} = 0.1 V<br>V_g = 0 V	2014	[25]
		FL MoS_2 - 1L Gr (at zero bias)	1428	3.0	-	0.13 ms	V_{ds} = 0 V	2015	[26]
Impurity engineering / energy band engineering of MoS_2	Phototransisrors based on doped MoS_2	APTES doped FL MoS_2	10	4.75×10^3	4.47×10^9	-	V_{ds} = 5.0 V<br>$V_g - V_{th}$ = 4.0 V	2015	[27]
		chemically in-situ n-type doped CVD MoS_2	-	99.9	9.4×10^{12}	τ_r = 16.6 s<br>τ_f = 5.2 s	V_{ds} = 0.1 V<br>V_g = 0 V	2019	[28]
	Phototransisrors based on energy band engineering of MoS_2	MoS_2 controlled by radiation pluses	-	0.0507	1.55×10^9	-	V_{ds} = 10 V<br>V_g = −15 V	2017	[29]
Gate engineering	Phototransisrors with local bottom-gate structures	Mechanically exfoliated ML	~ 10^3	342.6	-	>1 s	V_{ds} = 1.0 V<br>V_g = 8.0 V	2015	[30]
	Phototransisrors based on gate dielectric engineering	1-2L (HfO_2 encapsulation)	~ 10^3	10^4	7.7×10^{11}	10 s	V_{ds} = 1.0 V<br>V_g = −40 V	2015	[31]
		3L (driven by PVDF-TrFE)	10^3	2.57×10^3	2.2×10^{12}	τ_r = 1.8 ms<br>τ_f = 2.0 ms	V_{ds} = 5.0 V pre-polarized with V_g = −40 V	2015	[32]
		ML (inserting a TiO_2 interlayer)	> 10	9	-	< 1 s	V_{ds} = −1.0 V	2017	[33]
		ML MoS_2 ($Hf_{0.5}Zr_{0.5}O_2$ ferroelectric NC phototransistors)	3×10^6	10^2	4.7×10^{14}	τ_r = 400 μs<br>τ_f = 200 ms	V_{ds} = 0.5 V<br>V_g = −1.6 V	2019	This work

Supplementary Table. 1 | Optimization strategy and performance summary of reported MoS_2 phototransistors. ML: multilayer, FL: few-layer, CVD: chemical vapor deposition, NC: negative capacitance, QDs: quantum dots, Gr: graphene.

Supplementary References

[1] Muller J, et al. Ferroelectricity in simple binary ZrO_2 and HfO_2. *Nano Lett.* **12**, 4318-4323 (2012).

[2] Huan T D, et al. Pathways towards ferroelectricity in hafnia. *Phys. Rev. B* **99**, 064111 (2014).

[3] Sang X, et al. On the structural origins of ferroelectricity in HfO_2 thin films. *Appl. Phys. Lett.* **106**, 162905 (2015).

[4] Yin, Z. et al. Single-layer MoS_2 phototransistors. *ACS nano* **6**, 74-80 (2011).

[5] Choi, W. et al. High-detectivity multilayer MoS_2 phototransistors with spectral response from ultraviolet to infrared. *Adv. Mater.* **24**, 5832-5836 (2012).

[6] Lopez-Sanchez, O., Lembke, D., Kayci, M., Radenovic, A., Kis, A. Ultrasensitive photodetectors based on monolayer MoS_2. *Nat. Nanotechnol.* **8**, 497 (2013).

[7] Zhang, W., Huang, J. K., Chen, C. H., Chang, Y. H., Cheng, Y. J., Li, L. J. High-gain phototransistors based on a CVD MoS_2 monolayer. *Adv. Mater.* **25**, 3456-3461 (2013).

[8] Tsai, D. S. et al. Few-layer MoS_2 with high broadband photogain and fast optical switching for use in harsh environments. *ACS nano* **7**, 3905-3911 (2013).

[9] Perea-López, N. et al. CVD-grown monolayered MoS_2 as an effective photosensor operating at low-voltage. *2D Mater.* **1**, 011004 (2014).

[10] Lu, J. et al. Improved photoelectrical properties of MoS_2 films after laser micromachining. *ACS nano* **8**, 6334-6343 (2014).

[11] Ling, Z. P. et al. Large-scale two-dimensional MoS_2 photodetectors by magnetron sputtering. *Opt. express* **23**, 13580-13586 (2015).

[12] Lee, Y., Yang, J., Lee, D., Kim, Y. H., Park, J. H., Kim, H., Cho, J. H. Trap-induced photoresponse of solution-synthesized MoS_2. *Nanoscale* **8**, 9193-9200 (2016).

[13] Miao, J. et al. Surface plasmon-enhanced photodetection in few layer MoS_2 phototransistors with Au nanostructure arrays. *Small* **11**, 2392-2398 (2015).

[14] Lee, S., Park, J., Yun, Y., Lee, J., Heo, J. Enhanced photoresponsivity of multilayer MoS_2 phototransistor using localized Au schottky junction formed by spherical-lens

photolithography. *Adv. Mater. interfaces* **6**, 1900053 (2019).

[15] Kufer, D., Nikitskiy, I., Lasanta, T., Navickaite, G., Koppens, F. H., Konstantatos, G. Hybrid 2D-0D MoS_2-PbS quantum dot photodetectors. *Adv. Mater.* **27**, 176-180 (2015).

[16] Schornbaum, J. et al. Epitaxial Growth of PbSe Quantum Dots on MoS_2 Nanosheets and their Near-Infrared Photoresponse. *Adv. Funct. Mater.* **24**, 5798-5806 (2014).

[17] Chen, C. et al. Highly responsive MoS_2 photodetectors enhanced by graphene quantum dots. *Sci. Rep.* **5**, 11830 (2015).

[18] Huo, N., Gupta, S., Konstantatos, G. MoS_2-HgTe quantum dot hybrid photodetectors beyond 2 μm. *Adv. Mater.* **29**, 1606576 (2017).

[19] Yu, S. H. et al. Dye-sensitized MoS_2 photodetector with enhanced spectral photoresponse. *ACS nano* **8**, 8285-8291 (2014).

[20] Huang, Y. et al. Van der Waals coupled organic molecules with monolayer MoS_2 for fast response photodetectors with gate-tunable responsivity. *ACS nano* **12**, 4062-4073 (2018).

[21] Wang, Y. et al. Solution-Processed MoS_2/Organolead Trihalide Perovskite Photodetectors. *Adv. Mater.* **29**, 1603995 (2017).

[22] Kang, D. H. et al. An Ultrahigh-Performance Photodetector based on a Perovskite-Transition-Metal-Dichalcogenide Hybrid Structure. *Adv. Mater.* **28**, 7799-7806 (2016).

[23] Roy, K., Padmanabhan, M., Goswami, S., Sai, T. P., Ramalingam, G., Raghavan, S., Ghosh, A. Graphene-MoS_2 hybrid structures for multifunctional photoresponsive memory devices. *Nat. Nanotechnol.* **8**, 826 (2013).

[24] Zhang, W. et al. Ultrahigh-gain photodetectors based on atomically thin graphene-MoS_2 heterostructures. *Sci. Rep* **4**, 3826 (2014).

[25] Xu, H., Wu, J., Feng, Q., Mao, N., Wang, C., Zhang, J. High responsivity and gate tunable graphene-MoS_2 hybrid phototransistor. *Small* **10**, 2300-2306 (2014).

[26] Li, X., Wu, J., Mao, N., Zhang, J., Lei, Z., Liu, Z., Xu, H. A self-powered graphene-MoS_2 hybrid phototransistor with fast response rate and high on-off ratio. *Carbon* **92**, 126-132 (2015).

[27] Kang, D. H. et al. High-performance transition metal dichalcogenide photodetectors enhanced by self-assembled monolayer doping. *Adv. Funct. Mater.* **25**, 4219-4227 (2015).
[28] Li, S. et al. Enhanced performance of a CVD MoS_2 photodetector by chemical in situ n-type doping. *ACS Appl. Mater. interfaces* **11**, 11636-11644 (2019).
[29] Xie, Y. et al. Ultrabroadband MoS_2 photodetector with spectral response from 445 to 2717 nm. *Adv. Mater.* **29**, 1605972 (2017).
[30] Kwon, J., Hong, Y. K., Han, G., Omkaram, I., Choi, W., Kim, S., Yoon, Y. Giant Photoamplification in Indirect-Bandgap Multilayer MoS_2 Phototransistors with Local Bottom-Gate Structures. *Adv. Mater.* **27**, 2224-2230 (2015).
[31] Kufer, D., Konstantatos, G. Highly sensitive, encapsulated MoS_2 photodetector with gate controllable gain and speed. *Nano Lett.* **15**, 7307-7313 (2015).
[32] Wang, X. et al. Ultrasensitive and broadband MoS_2 photodetector driven by ferroelectrics. *Adv. Mater.* **27**, 6575-6581 (2015).
[33] Pak, Y. et al. Enhanced Performance of MoS_2 Photodetectors by Inserting an ALD-Processed TiO_2 Interlayer. *Small* **14**, 1703176 (2018).
